# Adhesion and Degranulation Promoting Adapter Protein (ADAP) Is a Central Hub for Phosphotyrosine-Mediated Interactions in T Cells

**DOI:** 10.1371/journal.pone.0011708

**Published:** 2010-07-22

**Authors:** Marc Sylvester, Stefanie Kliche, Sabine Lange, Sabine Geithner, Clementine Klemm, Andreas Schlosser, Arndt Großmann, Ulrich Stelzl, Burkhart Schraven, Eberhard Krause, Christian Freund

**Affiliations:** 1 Protein Engineering Group, Leibniz-Institut für Molekulare Pharmakologie (FMP), Berlin, Germany; 2 Fachbereich Biologie, Chemie, Pharmazie, Freie Universität Berlin, Berlin, Germany; 3 Institut für Molekulare und Klinische Immunologie, Otto-von-Guericke-Universität, Magdeburg, Germany; 4 Mass Spectrometry Group, Leibniz-Institut für Molekulare Pharmakologie (FMP), Berlin, Germany; 5 Institut für Medizinische Immunologie CCM, Charité - Universitätsmedizin Berlin, Berlin, Germany; 6 Otto-Warburg-Laboratorium, Max-Planck-Institut für Molekulare Genetik, Berlin, Germany; BMSI-A*STAR, Singapore

## Abstract

TCR stimulation leads to an increase in cellular adhesion among other outcomes. The adhesion and degranulation promoting adapter protein (ADAP) is known to be rapidly phosphorylated after T cell stimulation and relays the TCR signal to adhesion molecules of the integrin family. While three tyrosine phosphorylation sites have been characterized biochemically, the binding capabilities and associated functions of several other potential phosphotyrosine motifs remain unclear. Here, we utilize *in vitro* phosphorylation and mass spectrometry to map novel phosphotyrosine sites in the C-terminal part of human ADAP (486–783). Individual tyrosines were then mutated to phenylalanine and their relevance for cellular adhesion and migration was tested experimentally. Functionally important tyrosine residues include two sites within the folded hSH3 domains of ADAP and two at the C-terminus. Furthermore, using a peptide pulldown approach in combination with stable isotope labeling in cell culture (SILAC) we identified SLP-76, PLCγ, PIK3R1, Nck, CRK, Gads, and RasGAP as phospho-dependent binding partners of a central YDDV motif of ADAP. The phosphorylation-dependent interaction between ADAP and Nck was confirmed by yeast two-hybrid analysis, immunoprecipitation and binary pulldown experiments, indicating that ADAP directly links integrins to modulators of the cytoskeleton independent of SLP-76.

## Introduction

**Figure 1:**
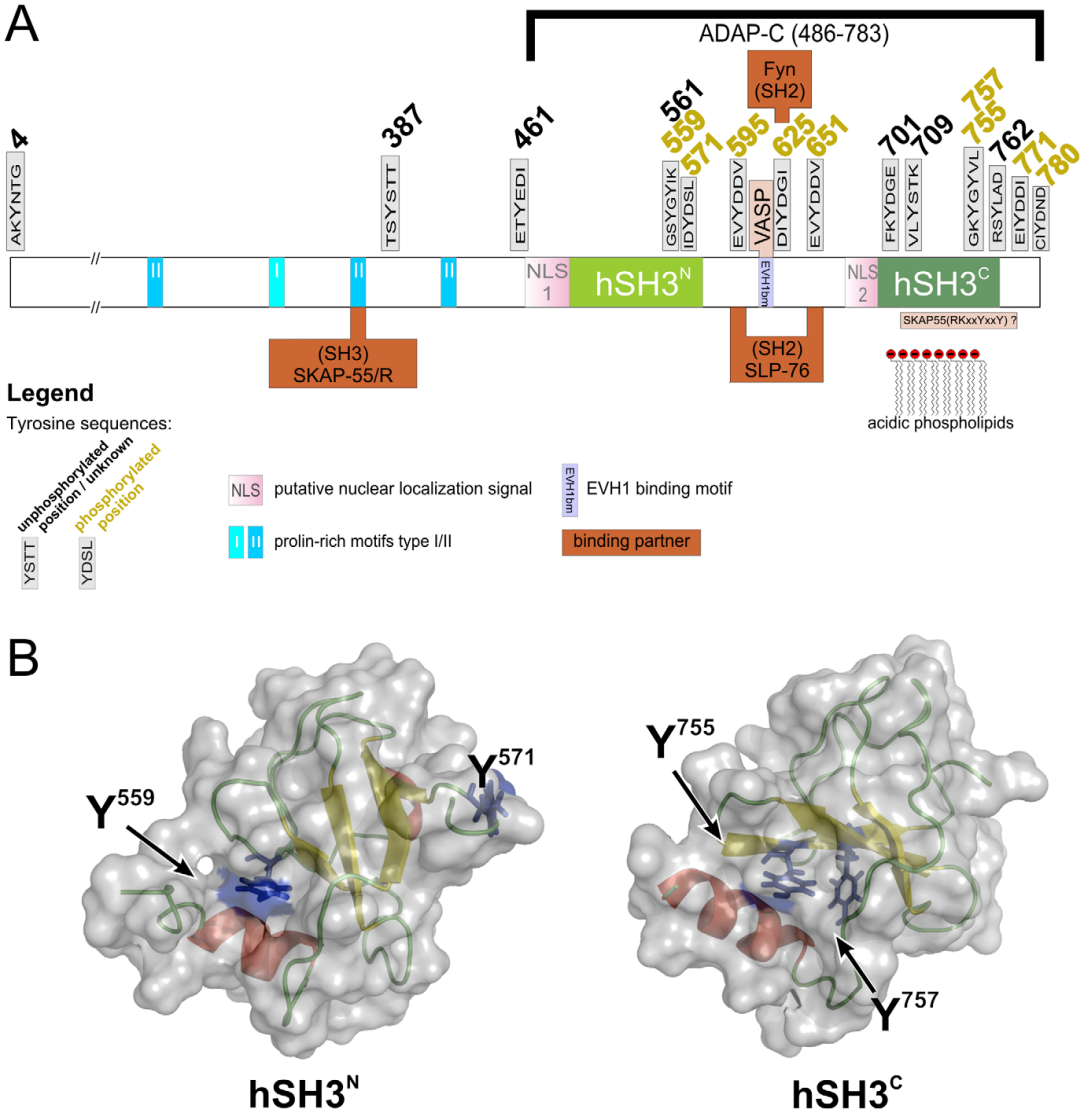
Sequence features of ADAP and three dimensional domain structures. **A** Schematic overview of ADAP primary structure indicating all tyrosine sequences, structured domains, binding partners, and major sequence features. **B** Three-dimensional representations of ADAP domains hSH3^N^ (left, reduced variant, PDB ID 2GTJ) and hSH3^C^ (right, PDB ID 1RI9). The surface is shown in transparent grey, secondary structure features are colored in red, green, and yellow. Residues that were found to be phosphorylated are shown with side chains in blue.

High affinity interactions between MHC:peptide complexes that match their clonotypic TCR lead to stable contact formation of antigen-presenting cells and T cells. Formation and maintenance of the “immunological synapse” rely on integrins, adhesion molecules that are indirectly regulated by TCR or chemokine receptor stimulation [1]. Tyrosine-phosphorylation of receptors and receptor-proximal signaling molecules lead to the recruitment of SH2 domain containing proteins that in turn transmit information to modulators of integrin activity. ADAP is one of the scaffolding proteins that are central to integrin activation and it is heavily phosphorylated at multiple tyrosines upon TCR stimulation [2], [3], [4], [5]. Most of the known and putative tyrosine-phosphorylation sites in ADAP are located within the C-terminal half of the protein that also contains two helically extended SH3 (hSH3) domains [6], [7]. The C-terminal hSH3 domain preferentially interacts with negatively charged lipids, while the function of its N-terminal hSH3 domain, apart from displaying a weak lipid binding affinity, is still enigmatic [8], [9]. ADAP constitutively associates with SKAP55 via a proline-rich domain in its N-terminal region. Fig. 1 summarizes the interaction motifs and domains of the protein as well as its known interaction partners. Three critical tyrosine motifs of ADAP are thought to coordinate the changes in protein assembly that accompany inside-out signaling. Interestingly, one of these sites (Y625) is assumed to bind to the Src family kinase Fyn, a kinase that can phosphorylate ADAP *in vivo* [10]. In addition to the Fyn binding site, two YDDV motifs are thought to be recognized by the SH2 domain of the adapter protein SLP-76 [10], [11]. SLP-76 in turn binds to the guanine-nucleotide exchange factor Vav1, the adapter protein Nck, and the Tec kinase Itk as well as to PLCγ1 and Gads. The constitutive interaction of Gads with SLP-76 recruits this complex to phosphorylated LAT, thereby linking the TCR to ADAP and integrin adhesion [12].

Simultaneous mutation of both YDDV motifs results in reduced cell conjugate formation and loss of LFA-1 polarization in stimulated Jurkat T cells [13], [14]. Despite the wealth of information on individual tyrosine phosphorylation motifs, two important questions remain unanswered: Can these motifs, upon phosphorylation, interact with other signaling molecules and secondly, are additional, functionally important tyrosine residues phosphorylated?

Here, we use *in vitro* phosphorylation to map tyrosine phosphorylation sites in ADAP (486–783) comprehensively. Mass spectrometric analysis reveals several sites of modification that comprise previously identified as well as novel sites. Two of these motifs are located in the folded hSH3 domains of ADAP at helix-sheet interfaces. Peptide pulldown experiments with a linear pYDDV-containing peptide show that several SH2 domain-containing proteins can bind to this motif in addition to SLP-76. Amongst these, actin cytoskeleton modulators Nck1 and Nck2 interact with the ADAP motif in a phosphorylation-dependent manner, thereby providing a direct link between integrin regulation and cytoskeletal rearrangements. Functionally, tyrosine to phenylalanine mutations of SLP-76/Nck interaction sites, of tyrosines in the hSH3 domains and in the C-terminus lead to an attenuation of Jurkat T cell adhesion and migration. This demonstrates that tyrosine phosphorylation of ADAP is more intricate than previously anticipated.

## Materials and Methods

### Antibodies

Antibodies were used for detection of phosphotyrosine (p-Tyr100, Cell Signaling Technologies, Inc., Danvers, USA), Fyn (CST, #4023), Nck (recognizing Nck1 and Nck2, Becton Dickinson GmbH, Heidelberg, Germany, #610099), ADAP (BD, #610945), and SLP-76 (Santa Cruz Biotechnology Inc., Santa Cruz, USA, #52789). For immunoprecipitation of ADAP sheep antiserum was used [15](kind gift of G. Koretzky). Secondary antibodies (donkey, highly cross-adsorbed) were AlexaFluor®680- or IRDye®800-labeled (Invitrogen Corporation, Carlsbad, USA and Rockland Immunochemicals Inc., Gilbertsville, USA respectively). For stimulation of Jurkat T cells OKT3 (eBioscience, San Diego, USA) or SDF-1α (Upstate Millipore GmbH, Schwalbach, Germany) were used.

### Constructs

ADAP constructs were derived from isoform2 (GenBank NM_199335.2). ADAP-C (aa 486–783), hSH3^N^ (aa 486–579), and hSH3^C^ (aa 683–768) were expressed in *E. coli* and purified utilizing a His_6_-tag or a GST-tag that was eventually removed. Tyrosine to phenylalanine mutants were created by PCR amplification of the complete pEFBOS vector containing ADAP cDNA using primers with a central missense mutation. GST-Nck1 SH2 and GST-Nck2 SH2 expression clones for in vitro binding assays were kindly provided by Gianni Cesareni and Bruce Mayer [16]. Nck1 and Nck2 in the Y2H experiments were full length ORFs (Nck1: aa 1–377, P16333; Nck2: aa 1–380, O43639). Full length ADAP and Fyn kinase (isoform 1) were purified from insect cells infected with Baculovirus carrying the corresponding His_6_ expression constructs.

### *In vitro* phosphorylation

Recombinant proteins (45 µM) were phosphorylated by incubation with Fyn kinase (2.3 µM) in 50 mM Tris·HCl, (pH = 7.5), 10 mM MgCl_2_, 0.5 mM EGTA, 0.1 mM sodium orthovanadate, 150 mM NaCl, 2 mM DTT, and 1 mM ATP at 20°C for 1 h. Tyrosine phosphorylation was detected after SDS-PAGE and western blotting with an anti phosphotyrosine antibody on the Odyssey® infrared imager (LI-COR Biosciences, Lincoln, USA).

### Data analysis

OdysseySoftware 2.1 (LI-COR) was used for quantification of phosphorylation signals. Statistical significance was tested with unpaired Student's t-test in Origin software. PyMOL was used for visualization of protein structures [17].

### Cell culture

Jurkat cells (clone E6-1, ATCC, or SLP-76-deficient clone J14 [18]) were cultured in RPMI1640 medium with 2 g/l NaHCO_3_, 2 mM ac-Ala-Gln (Biochrom AG, Berlin, Germany) supplemented with 10% FBS (Biochrom) without antibiotics in a humidified atmosphere with 5% CO_2_. For transfections 2×10^7^ cells were pelleted, washed and resuspended in 350 µl PBS containing Ca^2+^ and Mg^2+^ (Biochrom). After addition of 30 µg of DNA a 230 V, 950 µF pulse (high capacitance, Gene Pulser Xcell™, Bio-Rad Laboratories, Hercules, USA) was applied in 4 mm cuvettes (BTX-Harvard Apparatus Inc. Holliston, USA). Cells were cultured in a mixture of equal amounts of medium from passaged cells and fresh medium for 24 h before analysis. For stable isotope labeling by amino acids in cell culture (SILAC)[19] RPMI1640 medium deficient in Arg and Lys was used (Pierce® SILAC quantification kit, Thermo Fisher Scientific, Bonn, Germany) and supplemented with 10% dialyzed FBS, 0.1 g/l [^12/13^C_6_]L-lysine·2HCl, 0.2 g/l [^12/13^C_6_]L-arginine·HCl (Cambridge Isotope Laboratories, Inc., Andover, USA), and 2 mM L-glutamine (Invitrogen). Cells were cultured in SILAC media for nine population doublings.

### Adhesion and migration assays

Adhesion assays were performed as previously described [20]. Fibronectin (Roche Applied Science, Mannheim, Germany) and ICAM-1-Rg fusion protein were coated onto Falcon 1008 dishes. Where indicated in the figures, Jurkat T cells (10^6^ cells per dish) were incubated with OKT3 (TCR) for 30 min at 37°C prior to the adhesion assay. Cells were then allowed to adhere for 30 min at 37°C, unbound cells were carefully washed off with 3×1 ml Hanks buffered saline (HBSS, Biochrom) and bound cells were fixed with 3.5% paraformaldehyde in PBS. The bound cell fraction was determined with the aid of an ocular reticle. Migration assays [21] were performed using transwell® chambers with 5 µm polycarbonate filters (Costar®, Corning® B.V. Life Sciences, Schiphol-Rijk, The Netherlands) coated with 20 µg/ml fibronectin. Human SDF-1α (Tebu-Bio, Offenbach, Germany) was diluted to a concentration of 100 ng/ml in migration assay medium (RPMI supplemented with 1% bovine serum albumin fraction V, Roth, Karlsruhe, Germany) and 10 mM HEPES (pH = 7.4) and added to the lower chamber of the transwells. The fibronectin coated transwell inserts were placed on top and 2×10^5^ cells in 200 µl migration assay medium were added to the upper chamber. After 2.5 hours of incubation at 37°C, cells that had migrated through the filter were collected and counted.

### Enrichment of phosphoproteins

Phosphoprotein Purification Kit (Qiagen GmbH, Hilden, Germany) was used for affinity enrichment of phosphorylated proteins from *in vitro* phosphorylation for MS-analysis according to manufacturer's protocol except for elution which was achieved with 5×500 µl high phosphate buffer (20 mM PO_4_^3−^, pH = 7.3, 0.5 M NaCl).

### Protein coprecipitation assays

For immunoprecipitation 5×10^7^ Jurkat T cells were left unstimulated, stimulated with OKT3 (2 µg/ml in 2.5 ml serum-free medium) or SDF-1α (100 ng/ml) at 37°C for 5 min. Ice cold PBS was added to stop stimulation. Pelleted cells were lysed in lysis buffer (10 mM Hepes, pH = 7.5 at RT, 150 mM NaCl, 10 mM MgCl_2_, 10 mM KCl) with 1% (v/v) NP-40, 1 mM Na_3_VO_4_, protease inhibitor cocktail (complete, EDTA free, Roche Applied Science, Mannheim, Germany) on ice for 30 min. Cleared lysates were adjusted to [protein] = 5 g/l and incubated with ADAP or Nck antiserum (1∶150) at 4°C overnight. Immune complexes were precipitated with Protein-G-agarose (Sigma) for 2 h at 4°C and washed three times with TBS in centrifugal filter devices (Ultrafree-MC, Millipore). Bound proteins were eluted by boiling in SDS sample buffer.

Cosedimentation of purified Nck and ADAP was investigated by phosphorylation of purified ADAP constructs (4.5 nmol) with Fyn (0.23 nmol) for 1 h. 2.3 nmol of the phosphorylated ADAP protein were then incubated with GSH-sepharose coupled GST-Nck1/2 SH2 (1.6 nmol) for 2 h at 4°C. The matrix was washed three times with TBS and bound proteins were eluted by boiling in SDS sample buffer.

### Yeast two-hybrid experiments

Y2H experiments were performed as described previously [22]. In brief, the L40ccU2 MATa yeast strain [*MATa, his3Δ200,trp1-901, leu2-3,112, ade2, lys2-801am, GAL4, gal80, cyh2, can1, ura3::(lexAop)_8_-GAL1TATA-lac, LYS2::(lexAop)_4_-HIS3TATA-HIS3*] was co-transformed with a plasmid (pBTM117c) encoding the LexA-ADAP-C fusion and a plasmid (pASZ11-DM) encoding Fyn kinase domain (aa 251–520). Prey proteins were cloned as Gal4-AD fusions (pACT4-DM) and used to transform MATalpha strain L40ccα. Yeast strains were mated on YPD for 36 h. Diploid yeast were grown on SD media supplemented with histidine and uracil (SD2) for 3d. Interacting proteins were identified by growth on selective plates (SD4, -Leu-Trp-Ura-His, 20 µM CuSO_4_) and lacZ reporter gene activation assays after 5–7 days [23].

### Peptide pulldown experiments

Isotope labeled Jurkat T cells were washed three times with ice cold PBS before lysis (see above) for 30 minutes on ice. Cell debris was removed by centrifugation at 16,000×g at 4°C for 10 minutes. Lysates were used immediately for peptide binding assays. Peptides of the type CR^586^PIEDDQEV(p)YDDVAE^600^ were synthesized by standard solid-phase peptide synthesis (Fmoc chemistry). The N-terminal cysteine was attached to the ADAP sequence 586–600 to allow for covalent immobilization of the peptides on agarose beads using SulfoLink® Coupling Gel (Pierce) in accordance with manufacturer's instructions. Peptide binding assays were performed as duplicates in a crossover manner. Hence, each form of matrix-bound peptide (phosphorylated and non-phosphorylated) was incubated with either labeled or unlabeled cell lysates resulting in two independent pull down experiments. Incubation of 20 µl agarose beads (15 nmol peptide) was performed with 1.5 mg of total protein for 1 h at RT followed by four washing steps to remove unspecific binders. Bound proteins were eluted from the matrix with SDS sample buffer at 95°C for 5 min. Labeled and unlabeled samples were combined and proteins separated by PAGE on Tris-glycine gradient gels (4–20%).

The Coomassie-stained gel lane was cut into 40 slices. These were washed with 50% (v/v) acetonitrile in 50 mM ammonium bicarbonate, shrunk by dehydration in acetonitrile and dried in a vacuum centrifuge. The gel pieces were re-swollen in 10 µl of 50 mM ammonium bicarbonate containing 50 ng trypsin (sequencing grade modified, Promega). After 17 h of incubation at 37°C, 10 µl of 0.5% (v/v) trifluoroacetic acid in acetonitrile was added, samples were sonicated for 5 min, and the separated liquid was taken to dryness under vacuum. Samples were reconstituted in 6 µl of 0.1% (v/v) TFA, 5% (v/v) acetonitrile in water.

Peptides were analyzed with a reversed-phase LC system coupled to an LTQ-Orbitrap instrument. Details of LC-MS/MS procedures are provided as supporting text, File S1.

### Quantification of proteins

Quantification was carried out using the Mascot Distiller Quantitation Toolbox (version 2.2.1.2, Matrix Science) and was based on calculations of isotope intensity ratios of at least two arginine- or lysine-containing tryptic peptides with individual MASCOT scores indicating at least homology. Relative protein ratios were calculated from the intensity-weighted average of all peptide ratios. Proteins displaying enrichment factors >5 in two independent replicates were considered to be specific binding partners. Standard deviations of the quantification for individual proteins were obtained from Mascot Distiller. Analytical reproducibility was determined by multiple LC-MS measurements of selected samples showing that the experiment-to-experiment deviation of protein ratios is less than 25%.

### Identification of phosphorylation by mass spectrometry

Enzymatic in-gel digestion with either trypsin and AspN or multiple proteases followed by nanoLC-MS/MS analysis were performed with Q-TOF or MALDI-TOF mass spectrometers as previously described [24], [25], [26]. Data dependent acquisition was performed with preferential selection of peptides containing Tyr559, 561, 571, 701, 709, 755, 757, 762, 771, and 780. For details, see supporting text, File S1.

## Results

### ADAP phosphorylation analysis

Comprehensive mapping of tyrosine-phosphorylation sites in ADAP was achieved by *in vitro* phosphorylation of ADAP-C (aa 486–783) with Fyn kinase and mass spectrometric analysis. ADAP-C contains 13 of 16 tyrosines including the known phosphorylation sites, the two folded hSH3 domains, and all tyrosines with proposed functions (Fig. 1A).

Eleven of thirteen tyrosine residues could be analyzed by nanoLC-ESI and MALDI-MS/MS (Table 1). Three phosphorylation sites (Y559, 595, and 651) were identified using multiple proteases during MS sample preparation followed by phosphopeptide enrichment with titanium dioxide but could not be observed by using trypsin or AspN alone. Peptides containing Y561, 701, and 762 were found but only as non-phosphorylated species. Peptides containing the proposed Fyn kinase interaction site Y625 and Y709 were not observed in our MS experiments, while the known SLP-76 binding motifs comprising Y595 and Y651 were phosphorylated as expected. The C-terminal tyrosines at positions 780 and 771 were found to be phosphorylated, the latter one to a lesser extent (Table 1).

**Table 1:** Overview of phosphorylation data on all tyrosine and selected serine and threonine residues.

position	phosphorylation status/estimated level (this study)	literature
Y4	n.d.	phosphorylated in Jurkat T cells [34]
S46	n.d.	phosphorylated in platelets [35]
T158	n.d.	phosphorylated in platelets [35]
Y387	n.d.	
Y462	n.d.	
S558	phosphorylated*	
Y559	high	[3]
Y561	not phosphorylated	
Y571	high	Jurkat T cells [33], [34];U937 cells [36]; NSCLC-cell line [38], mouse mast cells [53], platelets [35], various other cell types: Phosphosite®, Cell Signaling Technology [http://www.phosphosite.org/])
S573	n.d.	phosphorylated [33], [34], [35], [53]
Y595	high	[10], [11]
Y625	n.d.	[10], [11]
Y651	high	[10], [11], Phosphosite (Jurkat)
Y701	not phosphorylated	
Y709	n.d.	
Y755	low	[33], Phosphosite (Jurkat)
Y757	low	[33], Phosphosite (Jurkat)
Y762	not phosphorylated	
Y771	low	Phosphosite (Jurkat), murine Y807, Src association [54]
Y780	high	Phosphosite (Jurkat)

n.d.: not determined, high/low: phosphorylation found, estimated level high/low, not phosphorylated: only data for unphosphorylated peptide was found. * Initial analysis of ADAP immunoprecipitated from activated Jurkat cells identified this site.

Importantly, two tyrosines in well-structured parts of the hSH3 domains were phosphorylated: Y559 in hSH3^N^ and Y755 in hSH3^C^. These residues are located at very similar sites in the three- dimensional structure at a contact area between β-sheet and N-terminal helix as shown in Fig. 1B. Both residues lie at the end of β-strand 4 and are partially buried by direct hydrophobic contacts to the helical residues I500 and F695, respectively. Preliminary NMR studies on phosphorylated hSH3^C^ indicated that the overall structure of the domain is intact after *in vitro* phosphorylation (data not shown). In addition, the tyrosine phosphorylation site at Y571 of hSH3^N^ that is located at the very end of the domain (Fig. 1B) was the most strongly phosphorylated residue (85% phosphorylation). The NMR ensembles (PDB ID 1RI9) for this region of the domain indicate a certain degree of flexibility and the side-chain of this tyrosine is fully solvent exposed.

We also examined the possibility of a positive feedback regulation of Fyn by phospho-ADAP because ADAP is a substrate for Fyn and subsequently becomes an interaction partner of the Fyn SH2 domain [10]. Binding of phospho-ADAP could release an inhibitory intramolecular binding of the SH2 domain and thereby foster its own phosphorylation. Thus, we monitored *in vitro* phosphorylation of ADAP-C by immunodetection of phosphotyrosines with varying kinase-to-substrate ratios but did not find significant differences (Supporting Figure S1). Under the conditions used in this study, there is no evidence for a positive feedback regulation of Fyn by ADAP-C.

### Functional aspects of tyrosine motifs

**Figure 2:**
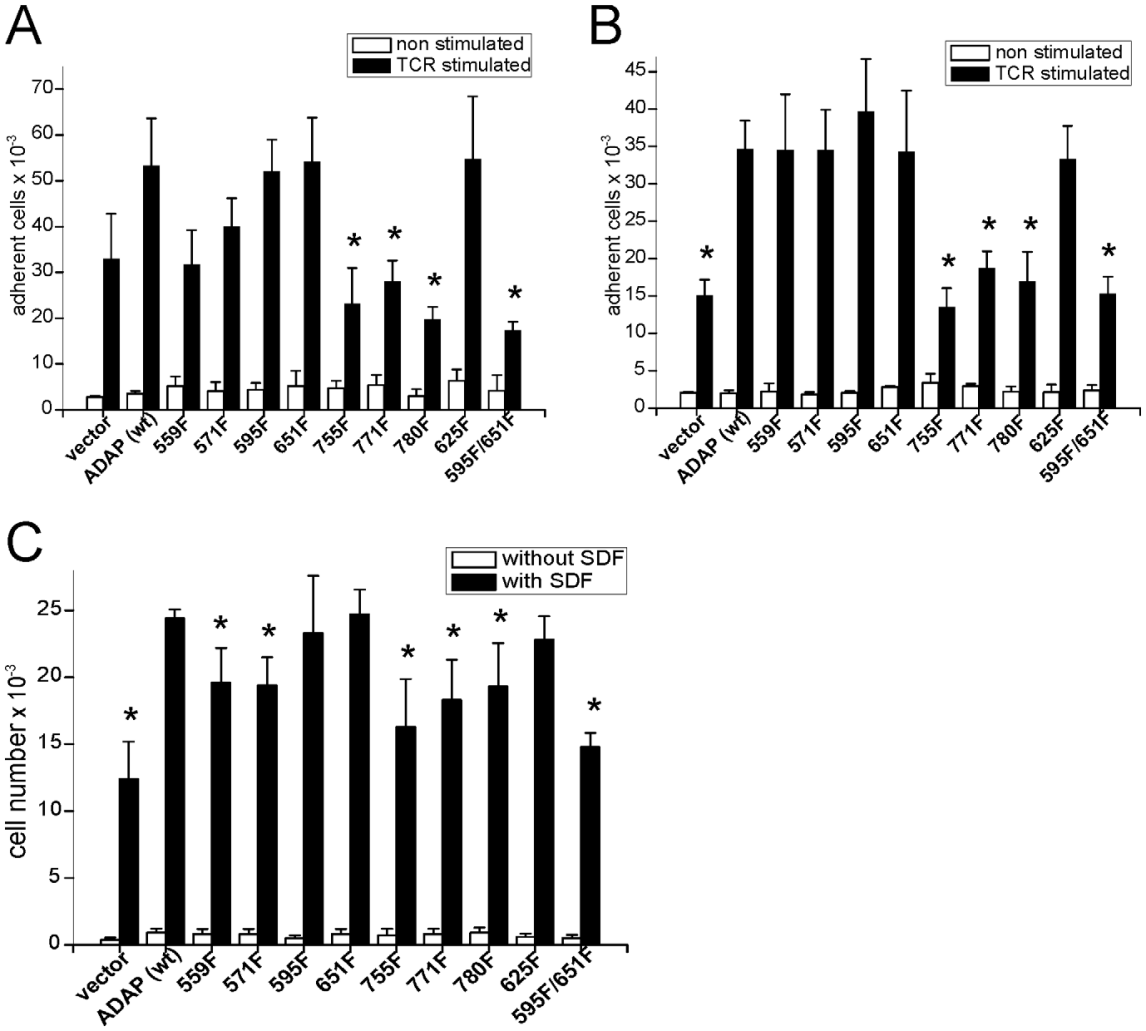
Cellular effects of tyrosine mutations. Mutations of ADAP tyrosines alter adhesion and migration of Jurkat T cells. **A, B** Adhesion assays of Jurkat cells on plates coated with fibronectin (A) or ICAM-1 (B) after overexpression of ADAP (wild type) or tyrosine to phenylalanine mutations at indicated positions. White bars: number of adhering cells without stimulation. Black bars: number of adhering cells after stimulation of cells with OKT3. **C** Migration of Jurkat cells through a transwell chamber in response to SDF-1. A-C: Average of absolute cell numbers from three experiments ± SD. “*” indicates significant deviation from stimulated ADAP (wild type) determined with Student's t-test.

To assess the functional importance of the identified phosphorylation sites, we created a set of single tyrosine to phenylalanine mutations in the context of full length ADAP, namely at positions 559, 571, 595, 625, 651, 755, 771, and 780. In addition, we prepared the double mutant Y595F/Y651F that was previously utilized by Geng et al. [11] to overcome a possible functional redundancy of the YDDV motifs. The individual constructs were overexpressed in Jurkat cells and tested for their ability to modulate integrin-dependent processes. Overexpression of wild type ADAP increases TCR-triggered LFA-1 adhesion to ICAM-1 about twofold as previously reported [13], [20] (Fig. 2B). This increase was statistically significant (p<0.05 in an independent Student's t-test). Mutating tyrosines 755, 771 or 780 abolished the adhesion promoting effect of ADAP overexpression, while T cells overexpressing ADAP with single mutations at positions 595 or 651 were indistinguishable from cells transfected with wild type ADAP. However, when both sites were mutated, ADAP failed to increase adhesiveness in agreement with previous reports [13]. Adhesion to fibronectin (Fig. 2A) showed comparable effects for all mutations with the exception of Y559F, which showed signal attenuation in this assay, while it displayed wild-type behavior for ICAM-1.

ADAP is also known to influence migratory properties of T lymphocytes positively [21], [27]. Hence, tyrosine mutants were tested for their ability to increase SDF-1-induced migration of Jurkat T cells. Phenylalanines at positions 559, 571, 755, and 771 reduced the pro-migratory effect of ADAP overexpression without completely abolishing it (Fig. 2C). The strongest effect was caused by the double mutation Y595F/Y651F while again, cells transfected with ADAP containing the corresponding single-site mutations migrated like wild type cells.

### Identification of phosphorylation dependent binding

**Figure 3:**
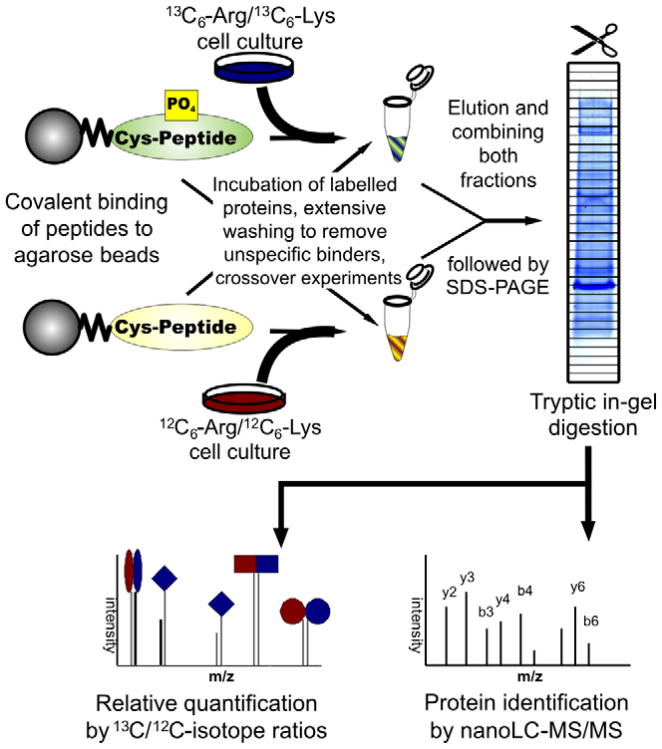
Peptide binding assay. Overview of SILAC based peptide binding assay. In our case, the ADAP peptide (586–600) was used either in its non-phosphorylated or tyrosine-phosphorylated form. Labeled or unlabeled Jurkat proteins were incubated with immobilized peptides on agarose beads. Bound proteins were separated by SDS-PAGE and identified by LC-MS/MS.

Phosphorylated ADAP motifs at Y595 and Y651 bind SLP-76 via its SH2 domain [10], [11]. Since the YDDV motifs are potentially recognized by SH2 domains other than SLP-76 we chose a peptide-based pulldown approach combined with SILAC methodology. Peptides containing phosphorylated or unphosphorylated Y595 were coupled to activated agarose and incubated with lysates from unstimulated Jurkat cells grown in media supplemented with either light or heavy amino acids ([^12/13^C_6_]lysine and [^12/13^C_6_]arginine, Fig. 3). All pulldowns were performed twice, in one experiment the ^13^C-labeled lysate was incubated with the phosphopeptide and the ^12^C-lysate with the non-phosphorylated peptide, while the mixing was reversed in the second experiment. After washing of beads, elution, and SDS-PAGE 1357 proteins were consistently identified by LC-MS/MS. SLP-76, PLCγ1, Nck1/2, RasGAP, Gads, PIK3R1, and CRK were specifically enriched by phosphopeptide in both experiments with most enrichment factors in the range of 40 or higher (Table 2).

**Table 2:** Enriched proteins after binding to immobilized phosphorylated compared to non-phosphorylated ADAP peptide around Y^595^ and MS analysis.

protein		replicate 1				replicate 2			
accession code	alternat. names	Mascot-Score	# peptides (ident)	^13^C/^12^C ratio	# peptides (quant)	Mascot-Score	# peptides (ident)	^12^C/^13^C ratio	# peptides (quant)
LCP2	SLP-76	387	32	>80	11	325	38	>80	9
NCK1		546	31	>40	15	563	37	>80	15
NCK2		377	21	>40	8	306	30	>80	8
P85A	PIK3R1	292	20	13.1	3	367	28	>40	5
PLCG1		1413	91	>80	36	1982	105	>80	44
RASA1	RasGAP	169	13	>40	3	240	17	>40	3
CRK		408	12	7.9	5	136	5	>40	2
GRAP2	GADS	178	12	>40	2	181	14	>80	4

**Figure 4:**
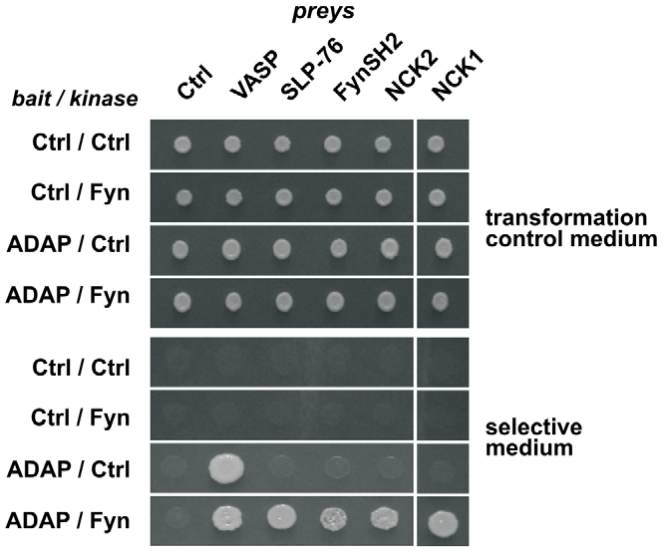
Nck coprecipitation with ADAP. Formation of a complex containing ADAP and Nck is increased after stimulation via TCR or chemokine receptor. Immunoprecipitation of ADAP from lysates of regular or SLP-76-deficient Jurkat T cells with a polyclonal antibody and detection of Nck after western blotting (upper panel). The antibody does not distinguish between Nck1 and Nck2. Detection of ADAP protein with a monoclonal antibody (center) and tyrosine phosphorylation (lower panel) on the same membrane. Data are representative for two experiments.

To test whether a complex between ADAP and Nck could be detected in cellular lysates, we performed immunoprecipitation experiments. Nck was found in a complex with immune precipitated ADAP from Jurkat T cells. The amount of coprecipitated Nck increased strongly after stimulation with OKT3 or SDF-1 (Fig. 4), in agreement with a stimulation-dependent phosphorylation of ADAP and a subsequent interaction with Nck. To further rule out the possibility that the interaction between ADAP and Nck is solely mediated by SLP-76, we performed experiments in SLP-76-deficient Jurkat cells. A small amount of ADAP-bound Nck was found in unstimulated Jurkat cells lacking SLP-76 while TCR-stimulation increased coprecipitation sharply in these cells. SDF-stimulation yielded the same amount of Nck in the absence or presence of SLP-76, indicating that chemokine stimulation renders the ADAP-Nck interaction independent of SLP-76 in both cell lines. Complex formation between Nck and ADAP followed the same tendency as ADAP phosphorylation, while the degree of Nck phosphorylation did not change significantly after stimulation (data not shown).

### Direct interaction of Nck and ADAP

**Figure 5:**
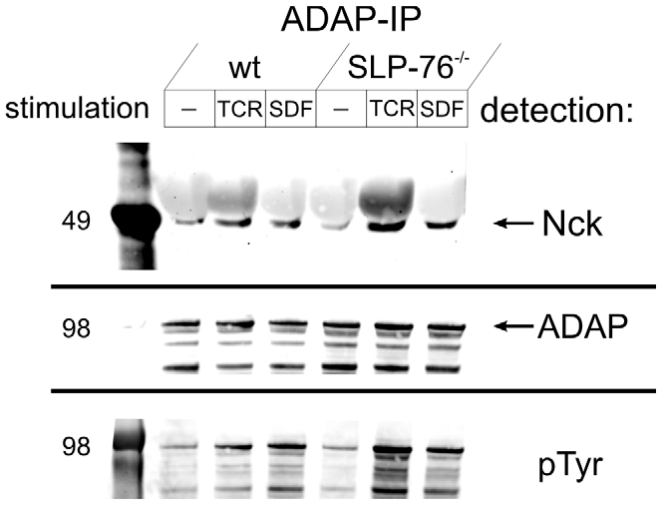
Kinase dependency of ADAP interactions. Analysis of phospho-dependent interactions in a modified Y2H system. Diploid yeast colonies (SD2-agar, top) expressing indicated bait, prey, and kinase constructs were assayed for growth on SD4-agar (bottom). Y2H interaction between ADAP-C and VASP is independent of Fyn kinase. In case of the SLP-76 (fl), Fyn SH2 (aa 151-246) and Nck1/2 (fl) preys, growth on SD4 is strictly dependent on the presence of active Fyn, indicating direct, phospho-dependent protein interactions.

**Figure 6:**
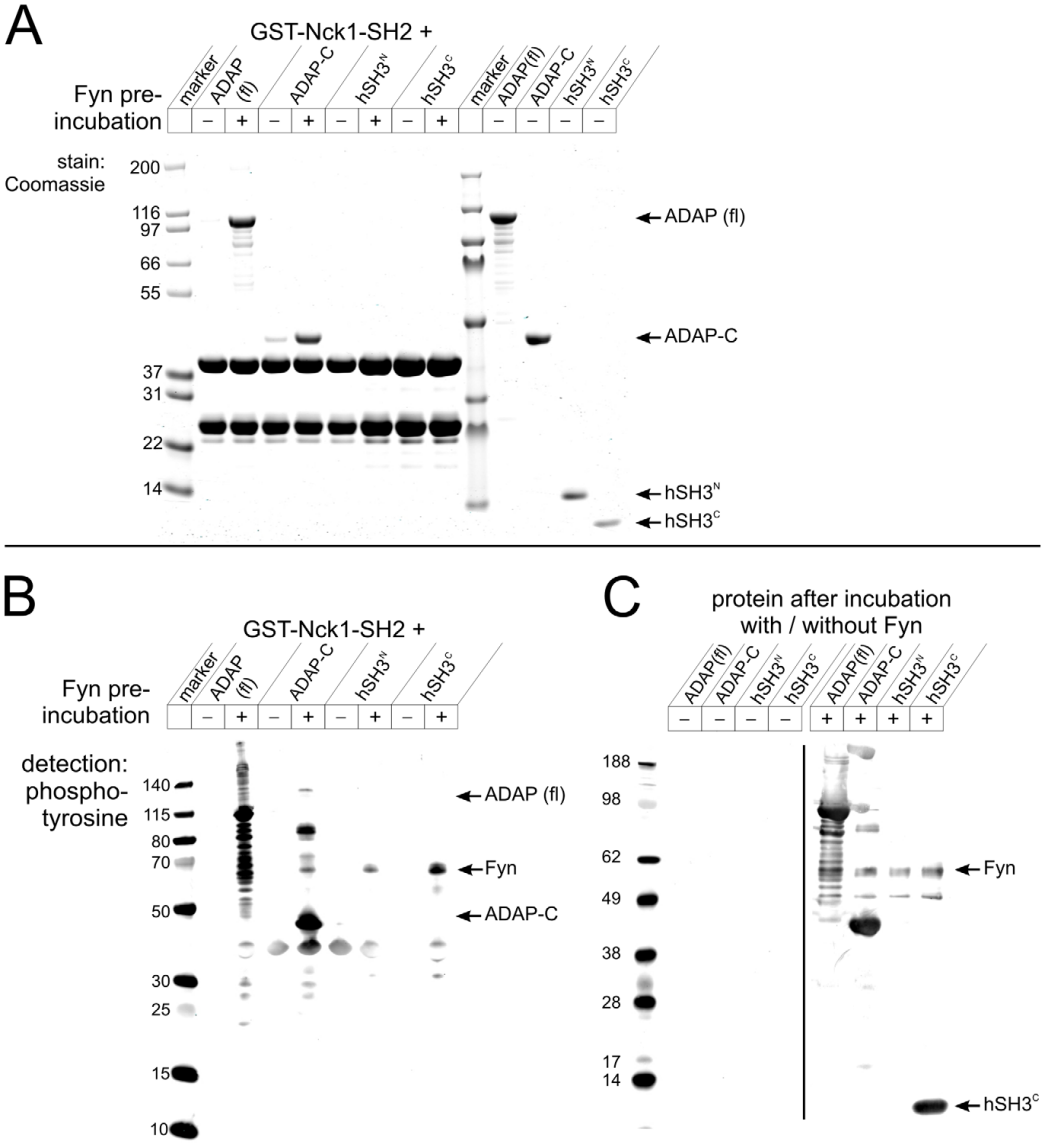
Direct binding of Nck to ADAP. Direct phosphorylation dependent interaction of ADAP and Nck SH2 *in vitro*. A *Left*: Purified ADAP (full length), ADAP-C, hSH3^N^ or hSH3^C^ were in vitro phosphorylated with Fyn as indicated. Subsequently, proteins were incubated with immobilized GST-Nck1 SH2. The washed GSH matrix was boiled in sample buffer, proteins separated by SDS-PAGE and stained with Coomassie. Band at approx. 24 kDa is GST as an impurity from GST-Nck SH2 preparation. *Right*: Protein flow through after pre-incubation (without Fyn) and subsequent incubation with GST-Nck1 SH2 to demonstrate protein stability and purity. B Phosphotyrosine detection after western blotting of same protein samples as in A. C Phosphotyrosine detection of protein samples after pre-incubation without or with Fyn as a control of phosphorylation by Fyn. hSH3^N^ phosphorylation was not readily detected by the antibody used.

Phosphorylated SLP-76 is known to bind the SH2 domain of Nck [28], [29]. Nck-SH2 binding predictions (SMALI) [30], [31] indicated that a direct recognition of the YDDV motifs of ADAP is probable. To test this possibility, yeast two-hybrid (Y2H) interaction analysis of ADAP-C with Nck1/2 and other ADAP interaction candidates was performed in the absence and presence of active Fyn kinase (Fig. 5). As expected, the EVH1 domain of VASP interacted independently of Fyn with ADAP-C [32]. Both interactions known to require ADAP phosphorylation, i.e. ADAP-C with SLP-76 and Fyn SH2, were recapitulated with high specificity. Interestingly, in our assay Y2H signals for interaction with full length Nck1 and 2 were strictly dependent on the presence of Fyn kinase, indicating a direct, phosphorylation-dependent interaction of ADAP-C with Nck. Other candidate proteins such as PIK3R1, RasGAP, CRK or Gads were tested but no colony growth was observed (data not shown). The directness and phosphorylation dependency of the ADAP-Nck interaction was further probed by pulldown experiments with recombinantly expressed and purified protein. Purified ADAP (full length) and ADAP-C bound immobilized GST-Nck1/2-SH2 while the isolated hSH3 domains could not (Fig. 6A, lanes 1–8). In addition, binding between ADAP and Nck was strongly enhanced if ADAP had been phosphorylated *in vitro* by Fyn kinase (Fig. 6B, lanes 3 and 5). Phosphorylation of Fyn itself and ADAP constructs is shown in Fig. 6C.

## Discussion

### Novel phosphorylation sites

In this report, we comprehensively describe tyrosine phosphorylation sites within the T cell protein ADAP and demonstrate their role for T cell adhesion and migration. Individual phosphorylation sites and their functional role had been described in Jurkat T cells [33], [34], platelets [35], U937 macrophages [36], and other cell types [37], [38] (Phosphosite, [http://www.phosphosite.org/]). Proteomic approaches in Jurkat cells, which relied on pervanadate treatment are in agreement with several of the sites defined in our study (Table 1). Our functional assays point to a novel role of tyrosines at position 559, 755, 771, and 780. Even single mutations of these residues lead to reduced augmentation of adhesion or migration after overexpression, demonstrating the non-redundant character of these tyrosines. Our results clearly show that phosphorylation at these sites has the potential to alter T cell adhesion. However, experiments utilizing primary cells will be required to show when and how this modulatory potential is utilized under more physiological conditions. For example, tyrosine phosphorylation at position 559 and the previously described phosphotyrosine 755 [33] map to regions within the folded hSH3 domains of the protein. Will phosphorylation at these sites change the foldability of the domains, expose the helix, alter lipid binding or create new sites for interaction partners? Initial experiments indicate that there is only a moderate decrease of binding affinity between liposomes and ADAP upon phosphorylation (data not shown). The electrostatic properties of the liposomes used in those experiments (PC:PS:PI(4,5)P_2_ = 54∶44∶2) resemble the charge distribution of the inner leaflet of the plasma membrane. Of course, a more dramatic influence of phosphorylation in the context of cellular lipid composition and membrane curvature cannot be ruled out but we believe that phosphorylation is not triggering membrane dissociation. More likely, phosphorylation of the YGY motif might attract novel binding partners. For SH2 domain binding to this motif, the domains would have to unfold (Fig. 1B), since the recognized peptide has to be linear and exposed within the context of at least four residues [39]. Preliminary NMR experiments indicate that phosphorylated hSH3^C^ maintains its fold (data not shown), however lipid binding might shift this equilibrium towards an unfolded conformation. Future experiments have to show the extent of structural changes imposed by phosphorylation and whether phosphorylation-dependent protein complex formation exists for hSH3^C^.

### The pYDDV motifs of ADAP as central interaction hubs

Strikingly, we find that the pYDDV motif selectively attracts proteins that take part in TCR proximal signaling which hints towards a function of this ADAP motif as a central hub for phosphorylation-dependent interactions. Six out of the seven highly enriched proteins found in our experiments are known constituents of the TCR proximal signaling complex and interestingly they were identified from a pulldown of unstimulated Jurkat cells. This indicates that the SH2 domains of these proteins are in a receptive conformation and are not blocked by intramolecular inhibition.

For the proteins listed in Table 2, direct interactions may be complemented by indirect interactions, since several proteins of the complex display binary interactions amongst each other. For example, Gads is known to bind constitutively to SLP-76 via a high-affine SH3-peptide interaction and could well be piggybacked by the latter in our pulldown experiments. Direct SH2 binding of Nck1/2 is predicted by the SMALI algorithm. Gads or SLP-76 SH2 domains yield smaller scores in this algorithm while the SH2 domain of PIK3R1 does not bind ADAP [40]. For PLCγ1 and RasGAP (N-terminal SH2) opposing predictions exist [31], [41]. Certainly, PLCγ and ADAP will be localized to the same membrane-proximal locale after receptor stimulation and PLCγ activity will lead to the production of diffusible IP_3_ and diacylglycerol. Neither of these two second messengers binds with significant affinity to the lipid-binding hSH3^C^ domain of ADAP (data not shown). In contrast, the educt of the reaction, phosphatidylinositol-(4,5)-bisphosphate, interacts with hSH3^C^ with a ∼20 µM K_D_ when assuming a simplified two-state binding model [6]. Therefore, a working hypothesis would be that PLCγ activity promotes spatially restricted membrane detachment of ADAP. Local production of PIP_3_ by PI3-kinase may counteract the depletion of PIP_2_ thereby keeping ADAP at the membrane at sites of high PIP_3_ concentration. However, whether such rearrangements and geometrical confinements are of relevance for cellular behavior remains to be investigated. The identification of RasGAP as a potential binding partner of ADAP raises the question whether there is a connection to the Ras signaling pathway or whether this molecule may act on Rap1 in the context of T cell signaling. The exchange factor RasGrp1 has been reported to bind to SKAP55, albeit the outcome of SKAP55 depletion in T cells remains controversial [42], [43]. However, RasGrp1 is a guanine nucleotide exchange factor acting on both Ras and Rap1, while RasGAP is specific for Ras. Nonetheless, RasGAP might still bind to Rap1, as it is suggested by the original finding of a signaling complex involving PLCγ, RasGAP and Rap1B that is important for platelet activation [44].

Since the amount of immobilized peptide is high, relatively low affine SH2:peptide interactions might be captured by our approach (Table 2). However, within the cell the local concentration of these SH2 domain-containing proteins could be high enough to result in simultaneous encounters of several binding partners with high off-rates. It is interesting to note in this context that pulldown experiments with the C-terminal homologous motif Y^771^DDI^774^ of ADAP resulted in the identification of the same interaction partners (data not shown), indicating that such a dynamic exchange of interactors might be of importance for complex formation.

### Direct interaction of ADAP and Nck

The experiments using SLP-76 deficient Jurkat cells, *in vitro* binding of Nck SH2, and the yeast two-hybrid experiments clearly argue for a direct interaction between ADAP and Nck. Recently, Lettau et al. identified the interaction of ADAP and Nck-SH2 in a screen for Nck binding partners [45]. In agreement with our observations, this interaction does not rely on SLP-76, which has a known pTyr recognition motif for the Nck SH2 domain. Early reports of SLP-76-Nck-binding already indicated the binding of a phosphoprotein of 120 kDa to Nck but this protein could not yet be identified as ADAP [28], [29]. ADAP and Nck had also been found to coprecipitate with Cbl (after SDF-1-stimulation [46]) or WASP [32]. Stimulation of Jurkat T cells enhanced the Nck-ADAP cosedimentation strongly but a weaker constitutive binding was observed by immunoprecipitation, presumably mediated by SH3-1 or SH3-3 of Nck [47] or by indirect binding. There are at least two instances where a direct interaction of Nck with ADAP might be of importance. First, there could be a structural requirement for the direct vicinity of ADAP and Nck. ADAP most probably interacts directly with the plasma membrane via its hSH3^C^ domain. This domain is 38 amino acids downstream of the second YDDV motif and would allow for a close proximity between the adapter and the membrane. This, in turn could promote the anchorage of the actin cytoskeleton at membrane attachment sites. Notably, there is an Ena/VASP/EVL interaction site in between the two YDDV motifs and VASP was previously described as colocalizing with ADAP at the T cell:APC junction and the membranes of the nascent phagosomes [48]. VASP was recently shown to induce processive filament elongation when functionalized on beads [49] and a similar function could be envisaged for the fraction of Ena/VASP/EVL that is drawn near the membrane via ADAP. Similarly, Nck would recruit other actin downstream effectors as for example N-WASP to the site of integrin delivery and activation. The modulating effect of ADAP on actin polymerization might be temporally and spatially restricted explaining the seemingly normal F-actin phenotype in cells from ADAP-deficient mice [2], [4]. It will be necessary to consider the direct link between Nck and ADAP in experiments where SLP-76 deficient cells or ADAP mutants lacking the YDDV motifs are analyzed.

### Orphan ADAP phosphorylation sites

Several of the other phosphorylation sites identified in our study might also harbor interesting functions. The motif around Y571 resembles an ITIM with predicted binding to CRKL, SHIP-2 or Tec SH2 domains but none of these interactions has been experimentally verified so far. Likewise, Y780 could represent a cognate motif for SH2 domains of T cell signaling proteins SH2D2A, HSH2D, and GRAP. The complexity of phosphorylation dependent signaling is further increased by parallel targeting of one protein by several kinases. For instance, a GPS2.0 based kinase prediction [50] identifies Tec, TXK and other candidate kinases for Y780 phosphorylation. Pyk2 is another kinase that is predicted to phosphorylate many ADAP tyrosine motifs (e.g. Y462, 595, 625, 771). Interestingly, immunoprecipitated Fyn from resting human T lymphocytes cosediments Pyk2, ADAP, SKAP55, and other proteins [51]. The complex is not found in resting Jurkat T cells, which points to differences between this cell line and primary cells. Lck is another important kinase in T cell signaling that is predicted to phosphorylate some of ADAP's tyrosines (e.g. Y595, 625, 651). The weak phenotype of Fyn^−/−^ T cells may be explained by a functional redundancy between Fyn and Lck [52]. However, the interaction between ADAP and Nck-SH2 could not be induced by Lck in our kinase-dependent Y2H experiments (data not shown). It will be necessary to investigate the role of this and other kinases targeting ADAP especially in the light of its overlapping functions in inside-out, chemokine, and outside-in signaling.

## Supporting Information

File S1 Supporting text.
(0.04 MB DOC)

Figure S1 Kinetics of ADAP phosphorylation by Fyn do not depend on substrate concentration. A ADAP-C (486-783) phosphorylation during incubation with Fyn. [Fyn] = 0.29 µM, molar ratios of ADAP as indicated. Time course of endpoint-normalized fluorescence intensities after Western blotting and immunodetection of phosphotyrosine. Average of three experiments ± SD. B Curve shape parameter a from non linear curve fitting plotted against substrate concentration. Results from individual results (open symbols) and average ± SEM.
(0.39 MB TIF)
