# Correction: Adhesion and Degranulation Promoting Adapter Protein (ADAP) Is a Central Hub for Phosphotyrosine-Mediated Interactions in T Cells

**DOI:** 10.1371/annotation/84d00e99-8566-4e44-b68f-a8d5b46db459

**Published:** 2010-08-18

**Authors:** Marc Sylvester, Stefanie Kliche, Sabine Lange, Sabine Geithner, Clementine Klemm, Andreas Schlosser, Arndt Großmann, Ulrich Stelzl, Burkhart Schraven, Eberhard Krause, Christian Freund

The images for figures 4 and 5 were incorrectly switched. The image that appears as figure 4 should be figure 5, and the image that appears as figure 5 should be figure 4. The figure legends appear in the correct order. 

