# Supplementary material for: Adhesion and Degranulation Promoting Adapter Protein (ADAP) Is a Central Hub for Phosphotyrosine-Mediated Interactions in T Cells
Source: PLoS One. 2010 Jul 22;5(7):e11708. doi: 10.1371/journal.pone.0011708 (PMC2908683; doi:10.1371/journal.pone.0011708)
Supplement: File S1 — Supporting text. (0.04 MB DOC) [file pone.0011708.s001.doc]

**Supporting text**

**Methods**

**Phosphorylation kinetics**

A kinase to substrate ratio of 2:1 to 1:10 was used with enzyme concentrations in the low µM range in 50 mM Tris·HCl, (pH=7.5), 10 mM MgCl_2_, 0.5 mM EGTA, 0.1 mM sodium orthovanadate, 150 mM NaCl, and 2 mM DTT. Components were mixed on ice, equilibrated to 20°C and the reaction was then initiated by mixing with ATP (1 mM final). Additional ATP was supplemented after 90 minutes in case of longer incubation times. Samples were taken and mixed with SDS sample buffer (including EDTA) at various time points to stop the reaction. Integrated and end point-normalized intensities of western blot bands were plotted on a logarithmic timescale. A non-linear least-square curve fitting procedure was performed (Origin®7.5, OriginLab Corporation, Northampton, USA) using the equation F=*a-b*·ln(*t*) where F represents normalized fluorescence intensity, *a* and *b* are curve shaping parameters and *t* is time. The parameter *a* is indicative of the initial slope of the curve.

## Enrichment of cellular ADAP

Lysates from 10^9^ pervanadate stimulated (10 µM, 15 min at 37°C) Jurkat cells were pre-cleared with Glutathione Sepharose™ 4B beads (GE Healthcare, Munich, Germany). GST-Fyn SH2 (isoform 1, amino acids 142-256) was immobilized on Glutathione Sepharose and incubated with the pre-cleared lysate (2.6 mg protein). Proteins were eluted from the washed matrix with SDS sample buffer at 70°C for 10 min.

**Mass spectrometric identification of peptide bound proteins**

LC-MS/MS analyses were performed on an LTQ-Orbitrap hybrid mass spectrometer (Thermo Fisher) equipped with an Eksigent 2D nanoflow LC system (Axel Semrau GmbH, Sprockhövel, Germany). The LC system was coupled to the LTQ via a nanoelectrospray source (Proxeon) with a 10 µm i.d. PicoTip ESI emitter (New Objective). 5 µl of the sample were injected and concentrated on a trap column (PepMap C18, 5 µm, 100 Å, 5 mm × 300 µm i.d., Dionex) equilibrated with 0.1% TFA, 2% acetonitrile in water. After switching the trap column in-line, LC separations were performed on a capillary column (Atlantis dC18, 3 µm, 100 Å, 150 mm x 75 µm i.d., Waters) at an eluent flow rate of 250 nl/min using a linear gradient of 0-40 % B in 50 min. Mobile phase A was 0.1% formic acid (v/v) in water, and B was 0.1% formic acid in acetonitrile. Mass spectra were acquired in a data-dependent mode with one MS survey scan (with a resolution 60,000) in the Orbitrap and MS/MS scans of the five most intense precursor ions in the LTQ. The MS survey range was m/z 350-1500. The dynamic exclusion time (for precursor ions) was set to 120 s and automatic gain control was set to 3 x 10^6^ and 20,000 for Orbitrap-MS and LTQ-MS/MS scans, respectively. The processed MS/MS spectra and MASCOT server (version 2.0, Matrix Science Ltd, London, UK) were used to search in-house against the UniProtKB/Swiss-Prot database (release 56.2). The maximum of two missed cleavages was allowed and the mass tolerance of precursor and sequence ions was set to 10 ppm and 0.35 Da, respectively. Acrylamide modification of cysteine, methionine oxidation, ^13^C_6_-isotope labeling of arginine and lysine were considered as possible modifications. A protein was accepted as unambiguously identified if the total MASCOT score was greater than the significance threshold and if at least two peptides appeared the first time in the report and were the top ranking peptides.

**Phosphorylation analysis by mass spectrometry**

Excised protein bands were reduced with 10 mM DTT and alkylated by replacing the solution with 55 mM iodoacetamide. After drying the gel pieces were reswollen in 10 µl of 5 mM NH_4_HCO_3_ containing 300 ng of either Trypsin (sequencing grade, Promega, Mannheim, Germany) or AspN (sequencing grade, Roche). 5 µl of 5 mM NH_4_HCO_3_ was added to keep gel pieces moist during enzymatic cleavage (37°C, overnight). Peptides were extracted by adding 10 μl of 0.5% trifluoroacetic acid in acetonitrile. The separated supernatant was dried under vacuum and redissolved in 6 µl of 0.1% (v/v) TFA in 10% acetonitrile-water (v/v). Peptides were purified with a C18 minicolumn ZipTipC18 (Millipore) according to manufacturer’s manual before analysis by MALDI-MS. Peptides were eluted with 5 µl of 0.3% TFA in water/acetonitrile (4:6 v:v). For Q-TOF analysis, the dried samples were redissolved in 6 µl 0.1% TFA in 10% acetonitrile (v/v). Alternatively, proteins were reduced with TCEP (10 mM) in sample buffer (10 min 95°C) and alkylated (50 mM iodoacetamide, 20 min room temperature) before SDS-PAGE. Digestions with trypsin, elastase, proteinase K, and thermolysin were performed overnight at 30°C in 0.1 M NH_4_HCO_3_ (pH=8). Extracted digests were combined, dried, and redissolved in 30% ACN, 2% FA. Peptides were loaded onto self packed titansphere nanocolumns. 0.1 M NH_4_HCO_3_ (pH=9) was used for elution, followed by formic acid treatment. NanoLC-MS/MS experiments were performed on a quadrupole orthogonal acceleration time-of-flight mass spectrometer Q-TOF Ultima or Q-TOF Micro (Micromass, Manchester, UK), equipped with a Z-spray nanoelectrospray source and a CapLC system or a nanoESI source, respectively. The spectrometers were operated in positive ion mode using PicoTip spray capillaries (New Objective, Woburn, USA). Typically, 5 µl of sample were injected using 0.1% TFA at a flow rate of 20 µl/min and concentrated on a precolumn (PepMap C18, 5 µm, 100 Å, 300-μm i.d. × 5 mm, Dionex, Idstein, Germany). Peptides were eluted onto an analytical column (PepMap 100, C18, 3 µm, 100 Å, 75-μm i.d. × 15 cm, Dionex) and separated using a 4-45% gradient of 0.1% formic acid in acetonitrile-water (8:2, v/v) against 0.1% formic acid (v/v) in acetonitrile water (5:95, v/v) in 100 min at a flow rate of 200 nl/min. Data dependent acquisition was performed with preferential selection of peptides containing Tyr559, 561, 571, 701, 709, 755, 757, 762, 771, and 780. MS/MS spectra (MassLynx version 4.0 software) were compared with theoretical fragment ions of proteolytic peptides of ADAP (486-783). Additional MS/MS experiments of selected peptides were performed on a MALDI-TOF/TOF instrument (4700 Proteomics Analyzer, Applied Biosystems, Framingham, USA) equipped with an Nd:YAG laser (355 nm). Peptide samples were purified on a C18 reversed-phase minicolumn as above and eluted with 5 µl 0.3% TFA in water/acetonitrile containing 10 μg alpha-cyano-4-hydroxycinnamic acid matrix. The MS/MS data were analyzed by use of Data Explorer software (Applied Biosystems). The degree of phosphorylation was estimated using ratios of the ion intensities (or ion currents) of phosphopeptides versus the corresponding non-phosphorylated peptides. Mascot Server (Matrix Science, London, UK) was used for database (SwissProt) searching.

**Results**

**Phosphorylation kinetics of ADAP.**

The possibility of a positive feedback regulation of Fyn by release of its auto-inhibition after phospho-ADAP binding was explored. We monitored *in vitro* phosphorylation of ADAP-C by immunodetection of phosphotyrosines with varying kinase-to-substrate ratios (Supporting Fig. 1A). Unlike classical enzyme kinetics, fast initial phosphorylation events are not monitored. Instead, the reaction is followed almost until endpoint in order to detect enzyme activity changes during the reaction.

Comparison of logarithmic curve fitting parameters did not show significant differences (Supporting Fig. 1B). We conclude that under the conditions used in this study, there is no evidence for a positive feedback regulation of Fyn by ADAP-C.
