# Supplementary figures and images for: Adhesion and Degranulation Promoting Adapter Protein (ADAP) Is a Central Hub for Phosphotyrosine-Mediated Interactions in T Cells
Source: PLoS One. 2010 Jul 22;5(7):e11708. doi: 10.1371/journal.pone.0011708 (PMC2908683; doi:10.1371/journal.pone.0011708)

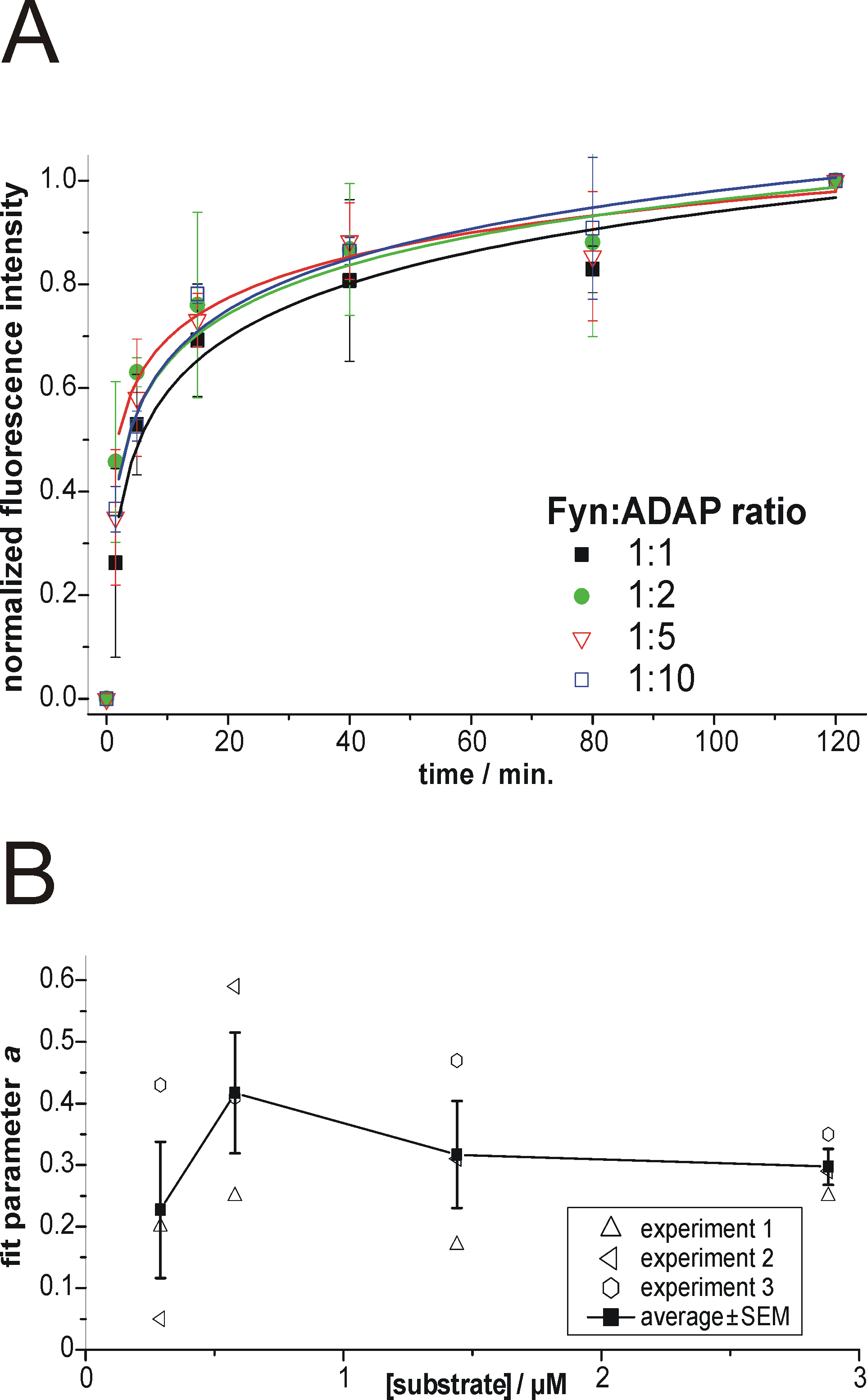

Supplement: Figure S1 — Kinetics of ADAP phosphorylation by Fyn do not depend on substrate concentration. A ADAP-C (486-783) phosphorylation during incubation with Fyn. [Fyn] = 0.29 µM, molar ratios of ADAP as indicated. Time course of endpoint-normalized fluorescence intensities after Western blotting and immunodetection of phosphotyrosine. Average of three experiments ± SD. B Curve shape parameter a from non linear curve fitting plotted against substrate concentration. Results from individual results (open symbols) and average ± SEM. (0.39 MB TIF) [file pone.0011708.s002.tif]
